# Training-Free Structural Damage Localization Using Spatial-Correlation Sensor Networks: Full-Scale Validation on a Seven-Story Reinforced-Concrete Building

**DOI:** 10.3390/s26175333

**Published:** 2026-08-23

**Authors:** Esmaeil Ghorbani, Jürgen Hackl

**Affiliations:** Department of Civil and Environmental Engineering, Princeton University, Princeton, NJ 08544, USA; ghorbani@princeton.edu

**Keywords:** structural health monitoring, damage localization, sensor networks, spatial correlation, random decrement technique, finite element updating, UCSD shear wall

## Abstract

Damage identification in instrumented structures is often framed through modal-parameter changes, finite element updating, or supervised classifiers. These approaches are powerful, but they require an explicit structural model, identified modes, labeled damage cases, or expert choices about reference sensors. This paper introduces a new data-driven and training-free approach with limited physical priors, defining a sensor network where each sensor is a node and the edges are defined from the spatial correlation of sensor responses. The idea is to use each sensor time history as the measured structural dynamics feature while damage is localized from the edges, whose correlations change relative to a baseline. The method is demonstrated on a full-scale seven-story reinforced-concrete shear-wall building tested at UC San Diego, considering four progressive earthquake-induced damage states and one brace-modification state. The results are compared with those obtained from a previously published finite element model. The results reveal that this network-based approach localizes the damage states in agreement with previous studies with limited prior requirements and low computational cost. Beyond damage localization, this network representation provides sensor centrality, allowing informative sensors to be selected from data rather than chosen randomly or only from experimental intuitions. For the case study, using this sensor network, we find the most central sensors, those carrying the most information with reduced trial-and-error and reduced expert intervention, and use them to recover the first three natural frequencies as a secondary dynamic check. The results show that spatial correlation networks can screen for damage, localize affected regions, and guide modal parameter extraction without building an FE model. This study opens a research avenue in which network representations of multi-sensor structural dynamics complement traditional modal analysis for structural health monitoring, with dense or heterogeneous sensing systems.

## 1. Introduction

Structural health monitoring (SHM) translates sensor data into actionable information about a structure’s condition, enabling risk-informed engineering decisions [1]. Damage identification generally involves three tasks: detecting the damage, determining its location, and quantifying its severity. Damage localization and quantification remain particularly challenging because they require a sufficiently expressive model whose accuracy may be compromised by uncertain inputs and parameters, including unknown excitation and operational variability, as well as by the curse of dimensionality. This paper represents the sensor data as a spatiotemporal correlation network, in which sensors are nodes and their spatial response correlations define the edges; damage is then localized by tracking edge changes over time, without requiring a mathematical model of the structure. The results are validated on a seven-story full-scale building and compared with the finite element model developed by Moaveni et al. [2].

In addition to visual inspection, classical vibration-based methods are widely used to identify structural damage through changes in global modal properties, including natural frequencies, mode shapes, curvature mode shapes, modal strain energy, and flexibility-based indices [3,4,5]. Although these methods can detect damage in the structure, reliable localization and quantification of damage remain difficult using these approaches [6]. Translating these global, environmentally sensitive modal shifts into localized physical property changes typically necessitates inverse formulations, such as finite element (FE) model updating [7]. FE model updating attempts to convert discrepancies between measured and predicted dynamic properties into physically meaningful estimates of stiffness, mass, damping, or boundary-condition changes [8,9]. This provides a direct connection between measured vibration data and structural parameters, but the inverse problem is often ill posed because multiple parameter combinations can produce similar results. Consequently, the inferred parameters depend strongly on the assumed FE model, selected updating parameters, boundary conditions, and objective function.

Bayesian model-data fusion methods, particularly Kalman filters, have been used to automate the FE model updating process and account for modeling and measurement uncertainties [10,11]. Although these methods enable quasi-real-time identification, allowing stiffness degradation or other damage-sensitive parameters to be estimated as measurements arrive, they require a reliable predictive state-space model, appropriate noise-covariance assumptions, sufficient sensor observability, and careful filter tuning. Their results may therefore remain sensitive to modeling and tuning assumptions rather than purely revealing the damage pattern [12,13].

In response to the limitations and difficulties of traditional methods for full-scale structures, statistical learning methods have been developed to extract damage-sensitive features directly from measured signals, images, or simulated structural responses [14,15]. For example, different techniques have been used for non-contact measurement, characterization of structural response, and damage detection, including phase-based methods for target-free three-dimensional deformation and laboratory-scale structural-displacement measurement [16,17], motion magnification for wind-turbine-blade damage detection [18], and computer vision for cable-force estimation from measured vibration and mode-shape information [19,20]. However, many of these approaches require large labeled datasets, simulated damage scenarios, careful domain adaptation, or expensive training procedures. Even when they achieve high accuracy, the learned features are often latent and difficult to interpret physically, or they fail to inherently account for the underlying spatial correlation and topological distribution of the sensor network [21,22].

Recently, graph signal processing (GSP) has received increasing attention for representing spatial correlations among sensor measurements [23,24], and recent SHM studies have used GSP and graph neural networks for sensor selection, missing-data reconstruction, spatiotemporal learning, and graph-based damage classification or localization [21,25,26,27,28]. In an earlier SHM application, Mohammadi Ghazi et al. [29] used pairwise probabilistic graphical models to combine information from dense sensor arrays, approximating graph parameters from mutual information and applying probabilistic inference to estimate intact or damaged states at individual sensor locations. By treating sensors as graph nodes and their spatial or physical relationships as edges, these methods can exploit correlations among measurements and support tasks such as sensor selection, missing-data reconstruction, damage classification, and localization. However, many existing graph-based SHM methods still depend on prescribed connectivity, supervised damage labels, simulated training cases, or learned representations that are not always easy to interpret mechanically. Therefore, although graph-based methods point toward network-aware SHM, there remains a need for a more transparent formulation that can be implemented directly from measured sensor responses and is not based on predefined connectivity.

The reviewed literature shows that existing SHM approaches provide powerful tools for damage identification, but their practical application often depends on modeling assumptions that introduce uncertainty in field settings. This leaves an important gap between global vibration features and local damage decisions, as extracting local information from global features often requires sufficiently expressive models [30]. This motivates an intermediate framework that does not require full FE model updating, does not rely on extensive training data, and does not treat the sensor network as a black box, but can still provide useful localization information. Accordingly, this study develops a sensor-network-based damage localization framework in which the structure is described through the measured interactions among sensors. Damage is identified by tracking how these sensor-to-sensor relationships change with respect to the reference condition. The proposed framework is computationally accessible and physically interpretable, while providing a foundation that can later be enhanced using more advanced uncertainty-aware, physics-informed, and graph-learning strategies.

This study integrates established correlation and network concepts into a transparent and training-free workflow for structural damage screening, and makes three contributions. First, a fully connected sensor network is inferred directly from pairwise response correlations, without prescribed connectivity or any training or learning stage; edge weight changes relative to the baseline then serve as interpretable localization indicators that are mapped to physical regions of the structure. On the benchmark, these indicators localize damage in agreement with the published FE-model-updating results across the four progressive damage states. Second, weighted-degree centrality computed from the same network identifies informative reference sensors for a secondary modal-frequency analysis, recovering the first three natural frequencies; damage localization and sensor selection are thereby linked within a single measured-response representation. Third, the framework is applied to a structural repair and rehabilitation state for which the previously calibrated FE parameterization provides no comparable damage factor, and the network indicators still reveal the associated response redistribution.

The proposed framework has three practical advantages. First, it uses only measured sensor responses and does not require a detailed FE model, supervised damage labels, or repeated training. In this benchmark, however, the final indicator is selected according to agreement with the FE reference, and mapping sensor-pair scores to FE substructures requires expert assignment. Second, it remains physically interpretable because the diagnostic quantity is based on measured sensor-to-sensor dependence rather than latent learned features. Third, it provides both localization information and sensor-importance information, which can support subsequent SHM analyses. At this stage, the method should be viewed as a complementary prior-light screening tool: it guides where to look, which sensor pairs carry localized damage information, and which individual sensors are useful as dynamic-response references.

The method is validated using a full-scale reinforced-concrete shear-wall benchmark with multiple earthquake-induced damage states previously characterized in the literature [2,31,32]. Section 2 describes the UCSD seven-story building benchmark and its damage states. The methodology is presented in Section 3, followed by the results in Section 4, and the discussion and conclusions in Section 5 and Section 6, respectively.

## 2. UCSD Benchmark and Damage States

**Figure 1:**
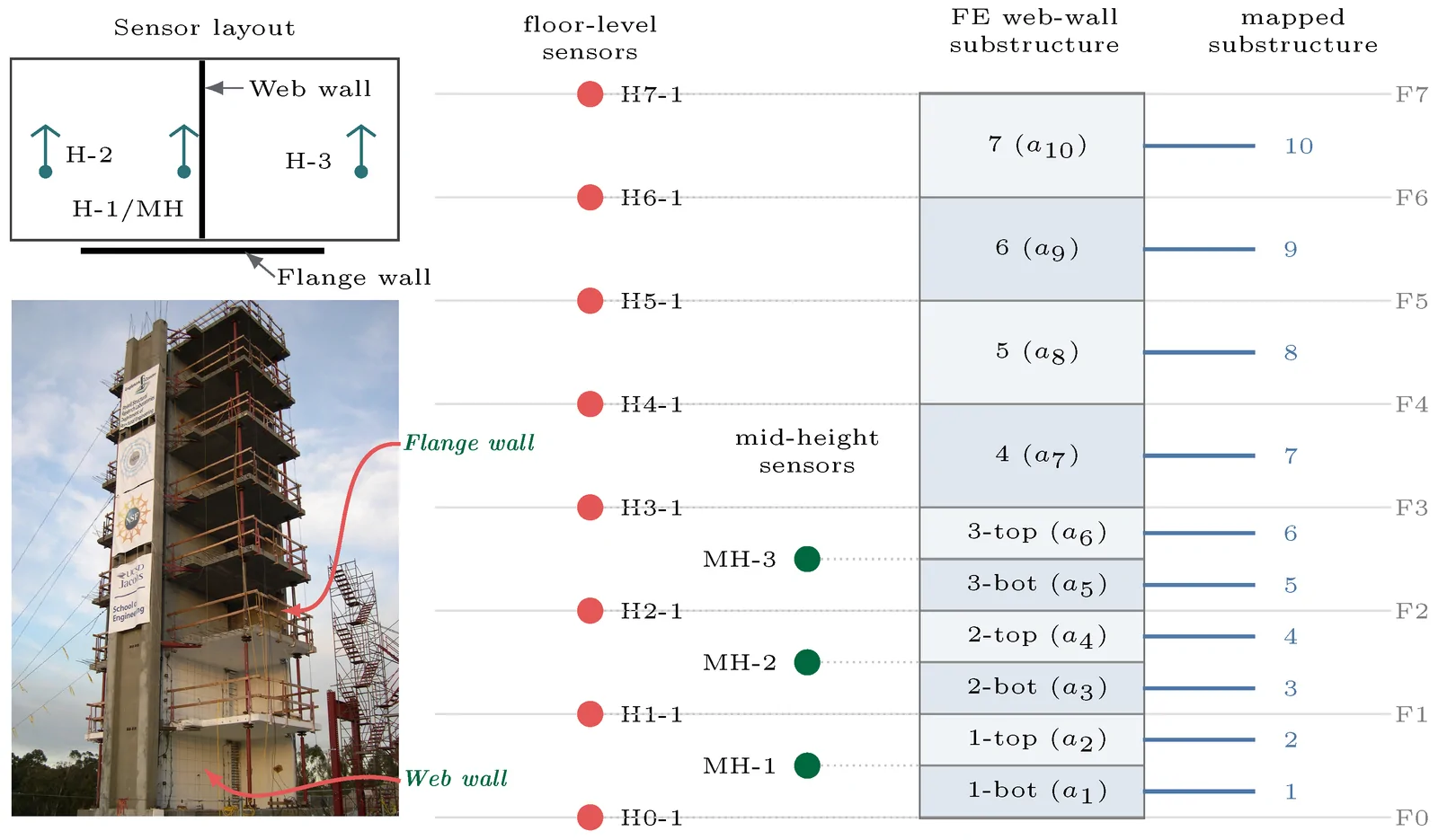
UCSD seven-story shear-wall specimen, longitudinal sensor layout, and substructure map. The network is built from the measured longitudinal accelerometer channels; for comparison with Moaveni et al.’s FE model results [2], selected sensor-pair scores are assigned to the ten web-wall substructures $a_{1}$–$a_{10}$. The rightmost numbers give the mapped substructure index used in the rank-based damage comparison. In each sensor label, the number after *H* denotes the floor level and the number after the hyphen denotes the sensor location on that floor. Green markers denote the three mid-height sensors, whereas red markers denote sensors located at the floor levels. Photograph source: [34].

To validate the proposed method, this study uses data from a seven-story reinforced-concrete building slice tested on the UCSD-NEES shake table, as shown in Figure 1. The specimen was first studied experimentally by Panagiotou [33]; Moaveni et al. [2,31] later used it for system and damage identification with an FE model updating technique. The data analyzed here correspond to Phase I of the test program, in which the primary lateral-force-resisting element is a rectangular web wall rather than a fully engaged T-wall. The specimen also includes a flange wall, transverse walls, floor slabs, gravity columns, and a post-tensioned column system, but the flange was deliberately not connected to the web wall as a load-bearing T-wall flange during Phase I [33].

The building was subjected to different damage states through a series of controlled earthquake excitations. The damaging inputs in the test program were four historical earthquake records applied in the longitudinal direction through the shake table. EQ1 used the longitudinal component of the 1971 San Fernando earthquake recorded at the Van Nuys station, while EQ2 used the transverse component of the same event and station. EQ3 used the longitudinal component of the 1994 Northridge earthquake recorded at the Oxnard Boulevard station in Woodland Hills, and EQ4 used the 360-degree component of the 1994 Northridge earthquake recorded at the Sylmar station [31]. S0 is the undamaged baseline before the first damaging earthquake. S1, S2, S3.1, and S4 are the states after successive earthquake inputs. Between S3.1 and S3.2, no new earthquake was applied; instead, the diaphragm-to-post-tensioned-column bracing was stiffened to maintain the desired second-mode frequency.

After each damage state, a low-amplitude white-noise base excitation over approximately 0.25–25 Hz, with an 8 min realization at an RMS amplitude of 0.03 g, was applied to excite the building. The vibration response was recorded at each floor, and the relevant test sequence is summarized in Table 1.

The local data files contain 45 acceleration channels sampled at 240 Hz; 29 longitudinal channels were used here to build the full correlation network and to compute sensor centrality because they measure the structural response in the direction of the applied shake-table excitation and the primary lateral response of the web-wall system considered in the damage-localization comparison. Channels measuring transverse or vertical responses were excluded to maintain a directionally consistent network and avoid combining responses governed by different structural mechanisms. Moaveni et al. [2] defined updating substructures along the web wall and tabulated damage factors for each. To compare the result with the published damage factors, following the same procedure, we defined ten substructures, labeled $a_{1}$–$a_{10}$: six along the first three stories (two per story—“bottom” and “top”) and four along the higher stories (one per story), as shown in Figure 1. However, Moaveni et al. used 11 substructures: the same ten web-wall regions plus one rotational spring at the base of the web wall that models shake-table flexibility and longitudinal-bar bond slip [2]. The proposed network was built using all selected longitudinal sensors, but the FE comparison used only the ten response intervals shown in Figure 1. These intervals map one-to-one onto Moaveni’s substructures definition $a_{1}$–$a_{10}$:

{1-bot,1-top,2-bot,2-top,3-bot,3-top,4,5,6,7},

adopting the same “bot”/“top” convention used in the benchmark study. This choice is not a sensor-selection step for constructing the graph; it is a validation step that puts the network score and the FE model result on the same vertical substructure coordinate.

The FE model includes an additional base rotational spring parameter, but this parameter does not correspond to a separate sensor-pair edge in the proposed network. In our network, the closest measured quantity is the H0-1–MH-1 edge, which spans the base-to-first-story region and is mapped to the 1-bot substructure in the ten-edge rank comparison. Because this edge is also sensitive to base-interface flexibility, we analyze its contribution separately in the base-sensor subsection. The upper mid-height sensors MH-4–MH-7 remain part of the full network and centrality analysis, but they are not used in the ten-edge FE comparison because Moaveni et al. do not subdivide the corresponding stories into “bot”/“top” substructures. With the benchmark, states, and validation map defined, the next section gives the graph quantities used to turn these sensor records into localization and sensor-importance measures.

## 3. Methodology

The hypothesis is that a structure’s spatial coordination of its dynamic response, as well as the way it distributes motion across sensors, is itself a measurable structural-dynamics feature, enabling damage detection and localization by tracking how this coordination changes across test states. This localization reading assumes quasi-stationary windows, broadband input, and dominance of lower longitudinal modes, under which local stiffness/interface changes are expected to perturb nearby sensor couplings more strongly than distant ones. Because global mode-shape redistribution can also change pairwise correlations, we treat mapped edge drops as a screening indicator, not as standalone proof of causality for any single edge.

**Figure 2:**
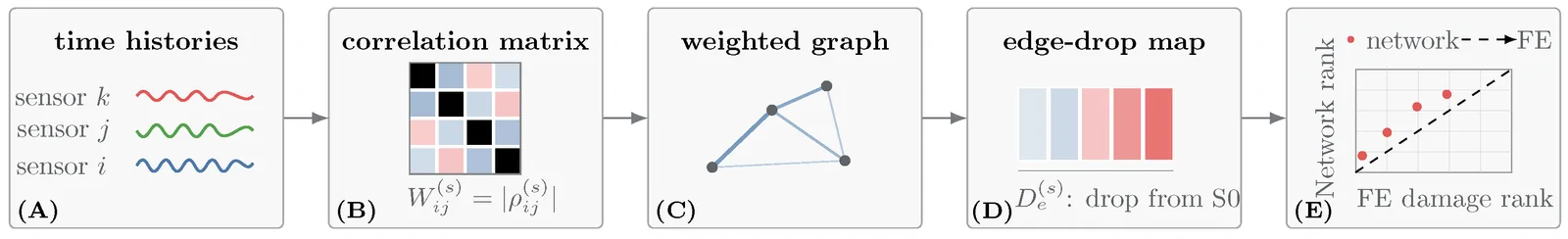
The correlation network workflow, shown from measured vibration data to rank comparison for validation. The measured records (**A**) define state-specific correlation matrices (**B**) and weighted sensor graphs (**C**); edge-weight drops relative to S0 (**D**) are mapped to the substructure intervals in Figure 1 and compared with the FE model damage pattern (**E**).

Figure 2 summarizes the complete workflow before the individual steps are defined. Starting from the measured vibration time history at each sensor (panel A), we compute the pairwise correlations between sensors and assemble them into a correlation matrix (panel B). This matrix is interpreted as the weighted adjacency matrix of a sensor graph, in which the nodes represent sensors and the edge weights are the absolute pairwise correlations (panel C). For each damage state, the drop in each edge weight relative to the undamaged baseline S0 is mapped onto the wall substructures (panel D) and ranked against the published FE-model damage factors of Moaveni et al. [2] (panel E) for validation. The remainder of this section formalizes each block of Figure 2 in turn: the correlation choices that define the edge weight, the substructure-edge map and damage score, the centrality-based sensor selection, and the statistical comparison with the FE model’s damage factors.

### 3.1. Correlation-Based Graph Construction

Let $x_{i}^{\left(s\right)}\left(t\right)$ be the vibration response of sensor $i\in \{1,\ldots ,n_{s}\}$ in state *s*. For the seven-story building considered here, $n_{s}=29$, corresponding to the number of longitudinal channels. For a chosen pairwise statistical-dependence function r(·,·), the weighted graph in state *s* is

(1) $$W_{ij}^{\left(s\right)}=\left|r\;\left(x_{i}^{\left(s\right)},x_{j}^{\left(s\right)}\right)\right|,\;i\neq j.$$

For compactness, $W_{e}^{\left(s\right)}$ denotes $W_{ij}^{\left(s\right)}$ when the mapped substructure edge is e=(i,j). Absolute value is denoted by |·| and is used because the mode-shape sign changes and anti-phase motion can still indicate strong dynamic-coupling magnitude. Before computing pairwise dependence, each channel is pre-processed consistently across states (de-meaned and linearly detrended), and each white-noise analysis window is treated as approximately weakly stationary so the sample correlations represent state-specific coupling rather than transient drift. Using |r| is therefore intentional for magnitude-based coupling loss, but it makes the primary score insensitive to sign-flip-only changes; directional (signed) changes are analyzed separately for S3.2 in Equation (10).

In this study, we use Pearson product-moment correlation, Spearman rank correlation, and a mixed Pearson/Spearman average as statistical measures to build the correlation matrix [35]. Let $x_{i}={\left[x_{i}\left(1\right),\ldots ,x_{i}\left(N\right)\right]}^{T}$ be the *N*-sample vibration record of sensor *i*, and

(2) $${\bar{x}}_{i}=\frac{1}{N}\sum_{k=1}^{N}x_{i}\left(k\right),$$

be its sample mean. The Pearson product-moment correlation between sensors *i* and *j* is

(3) $$\rho_{P}\left(x_{i},x_{j}\right)=\frac{\sum_{k=1}^{N}\left[x_{i}\left(k\right)-{\bar{x}}_{i}\right]\left[x_{j}\left(k\right)-{\bar{x}}_{j}\right]}{\sqrt{\sum_{k=1}^{N}{\left[x_{i}\left(k\right)-{\bar{x}}_{i}\right]}^{2}}\sqrt{\sum_{k=1}^{N}{\left[x_{j}\left(k\right)-{\bar{x}}_{j}\right]}^{2}}}.$$

Let $r_{i}\left(k\right)=\mathrm{rank}\left(x_{i}\left(k\right)\right)$ denote the rank of sample $x_{i}\left(k\right)$ among the *N* samples of $x_{i}$, with tied values assigned their average rank, and let $r_{i}={\left[r_{i}\left(1\right),\ldots ,r_{i}\left(N\right)\right]}^{T}$. The Spearman rank correlation is the Pearson correlation between the rank-transformed records:

(4) $$\rho_{S}\left(x_{i},x_{j}\right)=\rho_{P}\left(r_{i},r_{j}\right).$$

The three corresponding non-negative graph edge weights are

(5) $$\begin{array}{cc} W_{ij}^{\mathrm{Pearson}} & =|\rho_{P}\left(x_{i},x_{j}\right)|, \end{array}$$

(6) $$\begin{array}{cc} W_{ij}^{\mathrm{Spearman}} & =|\rho_{S}\left(x_{i},x_{j}\right)|, \end{array}$$

(7) $$\begin{array}{cc} W_{ij}^{\mathrm{mixed}} & =\frac{1}{2}\;\left(W_{ij}^{\mathrm{Pearson}}+W_{ij}^{\mathrm{Spearman}}\right), \end{array}$$

where $W_{ij}$ is the edge weight between sensors *i* and *j*. A separate symbol ρ (without the subscript) is reserved for the Spearman rank correlation that measures agreement between the network damage scores and the FE model damage factors across the ten mapped substructures $a_{1}$–$a_{10}$; the two uses are kept notationally distinct.

### 3.2. Damage Localization

For each mapped substructure edge e=(i,j), we score damage as a one-sided loss of correlation relative to S0. The primary score is the percent drop

(8) $$D_{e}^{\left(s\right)}=100\max\left(0,\frac{W_{e}^{\left(0\right)}-W_{e}^{\left(s\right)}}{W_{e}^{\left(0\right)}}\right).$$

and the sensitivity score is the Fisher-transformed drop

(9) $$Z_{e}^{\left(s\right)}=\max\left(0,\mathrm{atanh}W_{e}^{\left(0\right)}-\mathrm{atanh}W_{e}^{\left(s\right)}\right),$$

where atanh is the inverse hyperbolic tangent, $\mathrm{atanh}\left(x\right)=\frac{1}{2}\ln\;\frac{1+x}{1-x}$. The Fisher transform maps bounded correlations to an approximately unbounded scale with more stable sampling variance, so it is commonly used to compare correlation changes near high baseline correlations [36]. The method sweep reports $D_{e}^{\left(s\right)}$ as the “%” variant and $Z_{e}^{\left(s\right)}$ as the “z” variant.

To compare a network score vector with the FE model damage factors reported by Moaveni et al., we use the Spearman rank correlation ρ between the two length-10 vectors, one entry per mapped substructure edge, because Spearman compares the ordering of substructures from most to least damaged, not the numerical magnitudes themselves. A value ρ=+1 means both methods produce identical orderings (perfect localization agreement), ρ=0 means no relationship, and ρ=−1 means opposite orderings. Ranks are used because the network scores and the paper’s stiffness-loss damage factors are different physical quantities; only their spatial ordering is meaningfully comparable. Because the damage factors contain repeated values, Spearman handles ties by assigning the average rank to tied entries, which compresses fine distinctions within a tied group: ρ certifies that the network puts damage in the correct region of the wall but cannot certify the relative damage magnitude inside a region where the paper assigns several substructures the same value. We therefore treat ρ as a localization statistic rather than a precision error measure.

### 3.3. Permutation Test for Non-Random Localization

Because the number of substructures (here, 10) is small, and they are compared with a group of reference values, we estimate the significance of ρ using a Monte Carlo permutation test rather than the standard analytical approximation. The network scores are randomly reassigned to the substructures $N_{\mathrm{perm}}$ times, and ρ is recomputed after each reassignment while keeping the reference ranking fixed; here in this case study, we used $N_{\mathrm{perm}}=200,000$. The resulting shuffled correlations represent the correlations expected by chance. The reported two-sided *p*-value is the fraction of shuffled cases whose absolute correlation is at least as large as the observed one. The small permutation *p*-value indicates that the observed rank agreement is unlikely to arise from a random assignment of network scores to substructures.

### 3.4. Robustness Checks

Two additional analyses bracket the remaining sources of uncertainty.

*(i) Digitization sensitivity.* To test how much ρ depends on the damage factor, every digitized damage factor is perturbed by an independent draw from U(−5,+5) percentage points, clipped to [0,100], and ρ is recomputed against the network scores. Repeating this 10,000 times yields a sampling distribution for ρ that propagates a ±5 pp uncertainty at every substructure. The reported confidence interval is the 2.5th–97.5th percentile range of that distribution; if this range remains strictly positive, the rank agreement is robust to digitization errors of this magnitude.

*(ii) Baseline edge-weight variability.* To test how much an undamaged edge weight can change just because of finite-sample noise, the time history is divided into small subintervals, which yields a probability distribution of the weights for each state; for instance, the 8 min S0 sensor data is split into 16 non-overlapping 30 s blocks, and the correlation matrix is recomputed independently on each block. This gives 16 samples of every edge weight under the undamaged condition. The 2.5th quantile of those samples defines a per-edge baseline-noise floor. A damaged-state edge whose weight falls below this floor is declared as exceeding the natural variability of the undamaged structure—a stronger detection claim than merely showing that the weight dropped relative to the full-window S0 mean.

The sensitivity of this comparison to the selected block length was evaluated using non-overlapping blocks of 10 s, 15 s, 20 s, and 30 s. For these four block lengths, respectively, the numbers of mapped edges below the corresponding S0 lower bound were (2,2,2,3) for S1, (6,6,6,6) for S2, (6,6,6,6) for S3.1, and (3,4,4,4) for S4. Thus, the edge counts for S2 and S3.1 were unchanged over the tested range, whereas those for S1 and S4 varied by only one edge. These results consistently indicate changes across more mapped edges in S2 and S3.1, while the S4 response was concentrated in fewer edges. The 30 s blocks were retained for the reported baseline comparison.

### 3.5. State-Transition Diagnostic for S3.2

For the S3.2 brace-change state, we additionally use the signed edge change between two states *s* and $s^{\prime }$:

(10) $$\delta_{e}^{\left(s\to s^{\prime }\right)}=W_{e}^{\left(s^{\prime }\right)}-W_{e}^{\left(s\right)}.$$

Equation (10) differs from Equation (8) in two ways. First, it is not anchored to S0; it measures change between any two states. Second, it is signed, so a positive value (correlation strengthening, e.g., after a brace-induced coupling increase) is distinguishable from a negative value (correlation weakening, the signature exploited for damage localization). Although $\delta_{e}^{\left(s\to s^{\prime }\right)}$ can be computed for any pair of states, here it is used specifically for the transition from S3.1 to S3.2 because that transition corresponds to brace stiffening rather than a new earthquake input. For a state $s^{\prime }$ that follows pure damage progression relative to *s*, one expects $\delta_{e}^{\left(s\to s^{\prime }\right)}\leq 0$ on average across the mapped edges. A spatial mixture of $\delta_{e}>0$ and $\delta_{e}<0$ within the mapped edge set is therefore a coarse indicator that the S3.2 brace change is not behaving like a pure damage step.

The sign pattern of $\delta_{e}$ across edges, not the per-edge magnitude, is the discriminator we rely on for S3.2.

### 3.6. Centrality of the Graph

Using the network structure, we also compute secondary network quantities. First, $c_{i}^{\left(s\right)}$ denotes the thresholded weighted-degree centrality of sensor *i* in state *s*, used to identify sensors that remain strongly coupled to the global response:

(11) $$c_{i}^{\left(s\right)}=\frac{1}{n_{s}-1}\sum_{j\neq i}W_{ij}^{\left(s\right)}\;1\;\left(W_{ij}^{\left(s\right)}\geq \tau_{0}\right),$$

where $\tau_{0}$ is set to the 75th-percentile S0 edge weight; a sensitivity check over the 70th–90th percentiles preserves the principal centrality interpretation, with MH-6 and H5-1 remaining among the three most central S0 sensors and H0-1 retaining the largest S4 centrality loss. The indicator 1(·) is the indicator function, and the sum runs over the remaining $n_{s}-1=28$ sensors. Centrality itself is not a damage index: high centrality simply means that the sensor participates strongly in the dominant response. For localization, the meaningful quantity is the centrality change

(12) $$\Delta c_{i}^{\left(s\right)}=c_{i}^{\left(s\right)}-c_{i}^{\left(0\right)},\;$$

which is expected to be negative for sensors that lose coupling to the global response as damage progresses. Furthermore, the centrality is a valuable measure to select trigger or dominant sensors for further analysis. This is a separate role: it chooses sensors that are good references for extracting global modal frequencies, while the mapped edge drops provide the substructure localization. For instance, in the random decrement (RDT) application [37,38], the centrality-selected trigger sensors are used to compute the reference modal frequencies, and the mapped-edge drops are used to select which sensor pairs to use for the damage-sensitive RDT feature. Table 2 reports the highest-centrality sensors in the undamaged S0 state and the sensors with the largest centrality losses at S4.

**Figure 3:**
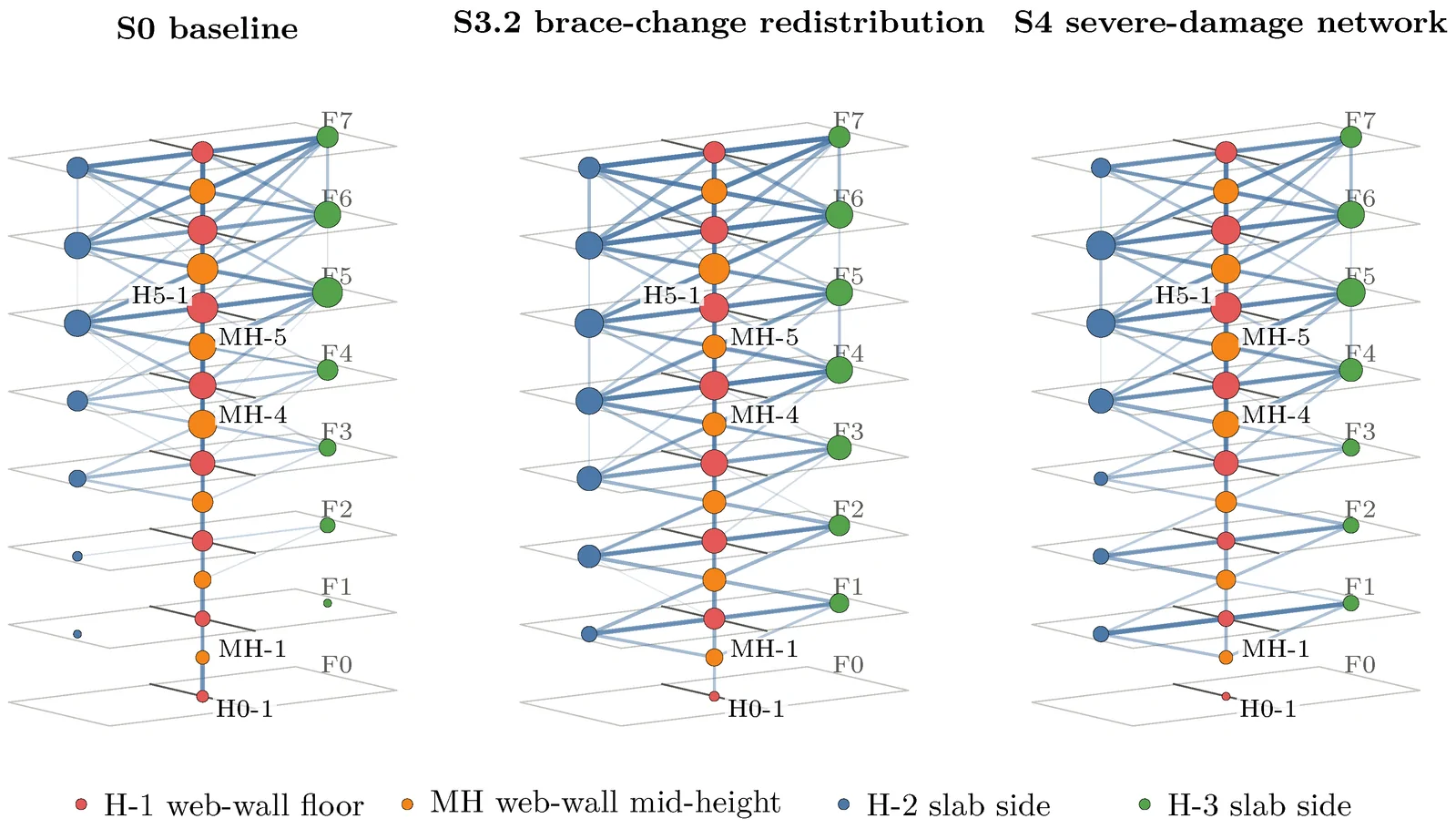
Spatial correlation maps of the sensor network at S0, S3.2, and S4. The sensor coordinates follow the plan layout of the UCSD specimen: H-2 channels are placed on the left slab side, H-3 channels on the right slab side, and H-1/MH channels on the web-wall line; the vertical coordinate is floor elevation. Colors distinguish these sensor families. Node size is the thresholded weighted-degree centrality $c_{i}^{\left(s\right)}$ computed from the full 29-channel longitudinal network, so larger nodes are sensors that remain strongly coupled to many other channels. Line width and opacity increase with the correlation weight. The three panels summarize the intended physical reading: the S0 baseline network is followed by a brace-change redistribution at S3.2 and then by the severe-damage network at S4.

Figure 3 is included as a geometric check on the network interpretation rather than as a separate damage metric. Edges are displayed when their absolute Spearman correlation is above the common 0.75 quantile of the three-state edge-weight pool (τ=0.851), and only the key sensors H0-1, MH-1, MH-4, MH-5, and H5-1 are labeled to avoid axis and grid clutter. This representation is used to avoid a possible misinterpretation of the graph: an edge denotes statistical coupling between two measured responses, not a physical wall or slab member. The network is first constructed from the experimental sensor layout and measured response data; selected edge scores are then associated with the corresponding FE model substructures for comparison with the published damage pattern. The S3.2 panel is retained because this state corresponds to a brace-induced redistribution rather than a nominal damage step, providing a visual reference for the signed-change analysis discussed below. At S4, the reduced connectivity near the base and lower web-wall region is consistent with the quantitative localization results, where the largest damage indicators occur in the bottom substructures.

## 4. Results

The results are organized to first select the best-performing correlation/scoring indicator, then to test whether the selected indicator localizes the damage pattern similar to the FE model, and finally to unpack why the useful correlations work, the spatial pattern, the base-sensor role, and the S3.2 brace-change state, all in the order of the Methodology section.

### 4.1. Indicator Selection

**Figure 4:**
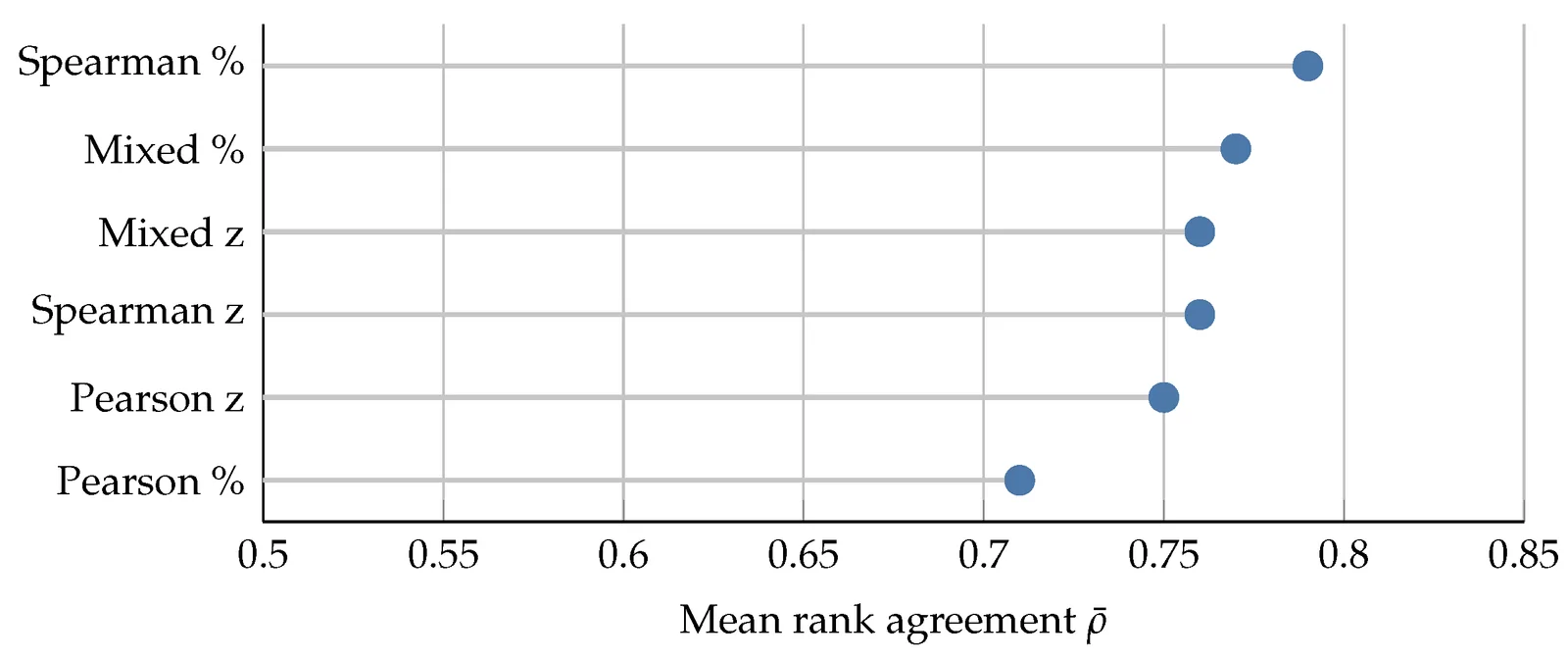
Comparison of mean rank agreement across S1, S2, S3.1, and S4 for the correlation/scoring variants. The selected indicator is Spearman absolute correlation with percent-drop scoring.

Figure 4 compares three correlation definitions, Pearson, Spearman, and their mixed average, each combined with two edge-drop scores: percent drop (Equation (8)) and Fisher-*z* drop (Equation (9)). For each of the six variants, we compute the Spearman rank agreement with the FE model damage pattern separately at S1, S2, S3.1, and S4, and then average those four ρ values to obtain the single plotted score $\bar{\rho }$. The highest mean rank agreement is obtained by Spearman absolute correlation with percent-drop scoring ($\bar{\rho }=0.79$), followed closely by the mixed and Fisher-*z* variants.

### 4.2. Damage Localization Performance

Table 3 is the numerical agreement summary for the selected indicator: it reports the state-wise rank correlations, permutation *p*-values, and the top-ranked substructures from the network and the paper reference. It gives strong rank agreement with the FE model damage pattern at S2, S3.1, and S4, although the top-three columns in Table 3 show that the agreement is regional rather than an exact recovery of the FE-updated ordering. The corresponding rank correlations are ρ=0.89, 0.94, and 0.86, with permutation *p*-values $9.4\times 10^{-4}$, $2.8\times 10^{-4}$, and $2.62\times 10^{-3}$, respectively; by contrast, S1 gives only ρ=0.47 with p=0.175. This is consistent with the benchmark paper, where the FE model cases and the visual inspection evidence do not coincide exactly but all point to dominant lower-wall damage. In particular, Moaveni et al. [2] report that based on visual inspection, the observed S4 damage is concentrated in the bottom two stories, while their FE result also contains a fourth-story damage component. They attribute this fourth-story component partly to the FE model parameterization absorbing damage effects from non-updated components and partly to increased S4 uncertainty caused by stronger nonlinear response and optimization sensitivity. The network-based indicator in this study uses the measured change in sensor-to-sensor spatial correlation directly, so it avoids this particular source of model-parameter compensation. For this S4 case, the network result is more consistent with the reported visual damage pattern: its three largest edge drops are 1-bot, 2-bot, and 1-top, all in the observed lower-wall damage region and without the fourth-story component. At S2 and S3.1, the network also localizes the response change to the lower wall, but one of the top-three edges shifts from 2-bot in the FE reference to 3-bot in the network result. S1 is weaker, which is physically plausible because the paper’s damage factors are small and tied for several substructures; the spatial coupling changes are close to the noise floor, and the method should be considered unreliable for this mild-damage state.

Because several candidate indicators were tested on the same damage states summarized in Figure 4, the reported *p*-values do not account for the selection of the best-performing indicator. The *p*-values should therefore be viewed as supporting evidence for the selected indicator, rather than as formal significance tests for a method chosen before examining the data. To check whether this selection made the performance appear overly favorable, a leave-one-state-out analysis was performed. Each damage state was excluded in turn, the indicator was selected using the other three states, and its performance was then evaluated on the excluded state. The mean correlation from this analysis was ρ=0.76, close to the value of ρ=0.79 obtained using all four states. This small difference suggests that the result is not strongly affected by the indicator-selection step. However, because only four damage states are available, this analysis is a robustness check rather than a fully independent validation.

The comparison is limited by the paper-damage reference because the FE model damage factors are read from a figure and include tied, rounded values. Thus, the reported rank correlations should be interpreted as coarse spatial agreement with the FE model pattern and the observed lower-wall damage region, not as a metrological comparison of damage magnitudes. In the benchmark, the visual inspection is treated as the primary physical reference, while the FE model damage factors are a secondary, model-dependent proxy; therefore, a high rank correlation is interpreted as agreement with the FE ordering, not as standalone proof of physical localization success.

Table 4 has a different role from Table 3: it checks whether the numerical agreement is stable to digitization uncertainty and whether the measured edge drops exceed the natural variability of the undamaged baseline. These robustness checks show that the digitization warning matters mainly for S1: when the digitized damage factors are perturbed by ±5 percentage points, the S1 rank agreement spans a wide interval, while S2, S3.1, and S4 remain strongly positive. Splitting S0 into sixteen non-overlapping 30 s blocks also shows that the largest S4 signal is not a baseline artifact: the 1-bot edge remains far below the lower S0 block-CI bound. In Table 4, the third column counts how many of the ten mapped substructure edges fall below this baseline-variability bound. The interpretation is that S2 and S3.1 are not driven by one isolated edge: in both states, six of the ten mapped edges exceed the S0 variability bound. At S4, four of the ten mapped edges exceed the bound, so the severe-damage result is still larger than baseline variability but more spatially concentrated. S1 remains weak because its digitization interval includes low positive agreement and only three of the ten mapped edges exceed the baseline bound.

### 4.3. Visualizing Rank Agreement and the Spatiotemporal Pattern

**Figure 5:**
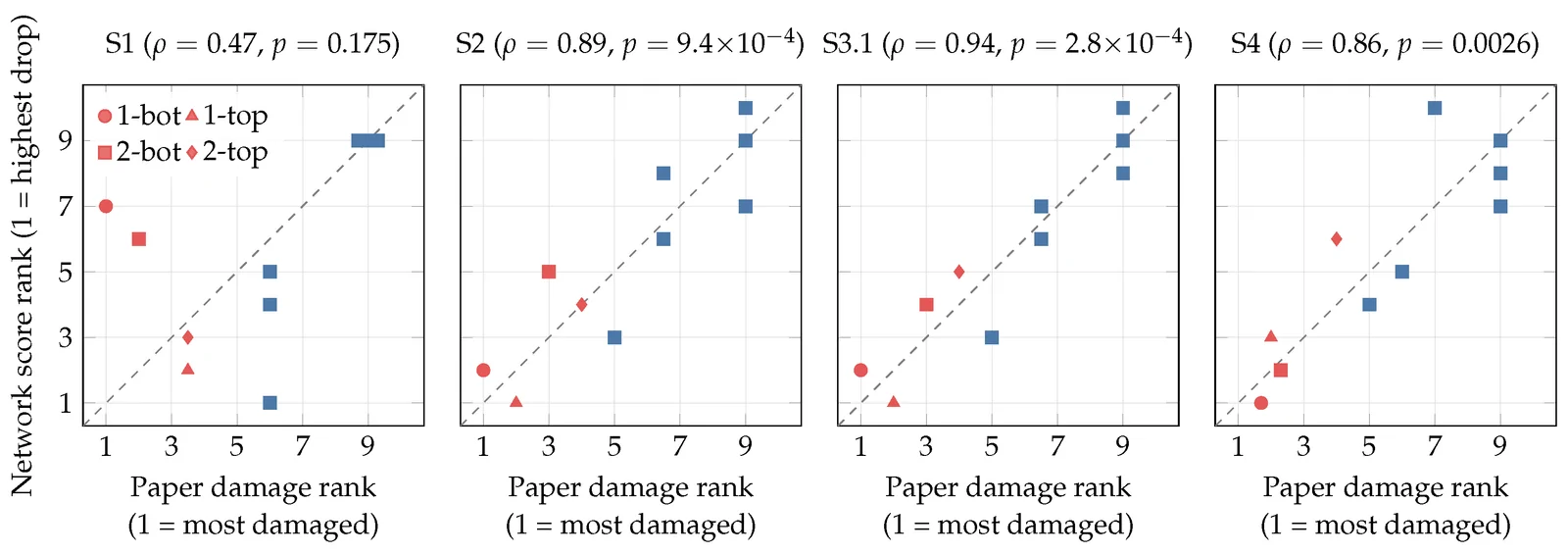
Rank–rank agreement between the network damage-localization score and the paper FE model damage factors, by state. Each marker is one of the ten mapped substructures; the four bottom-region substructures use the red marker shapes shown in the legend, and blue squares denote the remaining upper substructures (3-bot, 3-top, 4–7). Points on the dashed diagonal are perfectly co-ranked. Tied paper or network ranks are jittered slightly only for visibility; the plotted points therefore show rank agreement, not physical sensor or floor locations. The pattern is qualitatively different between S1 and the later states: at S1, the network places 1-bot at rank 7 while the paper places it at rank 1, and the paper’s least-damaged region 3-bot is the network’s most-affected edge (rank 1); from S2 onward, most lower-region markers move close to the diagonal, and the global rank statistic exceeds 0.85.

**Figure 6:**
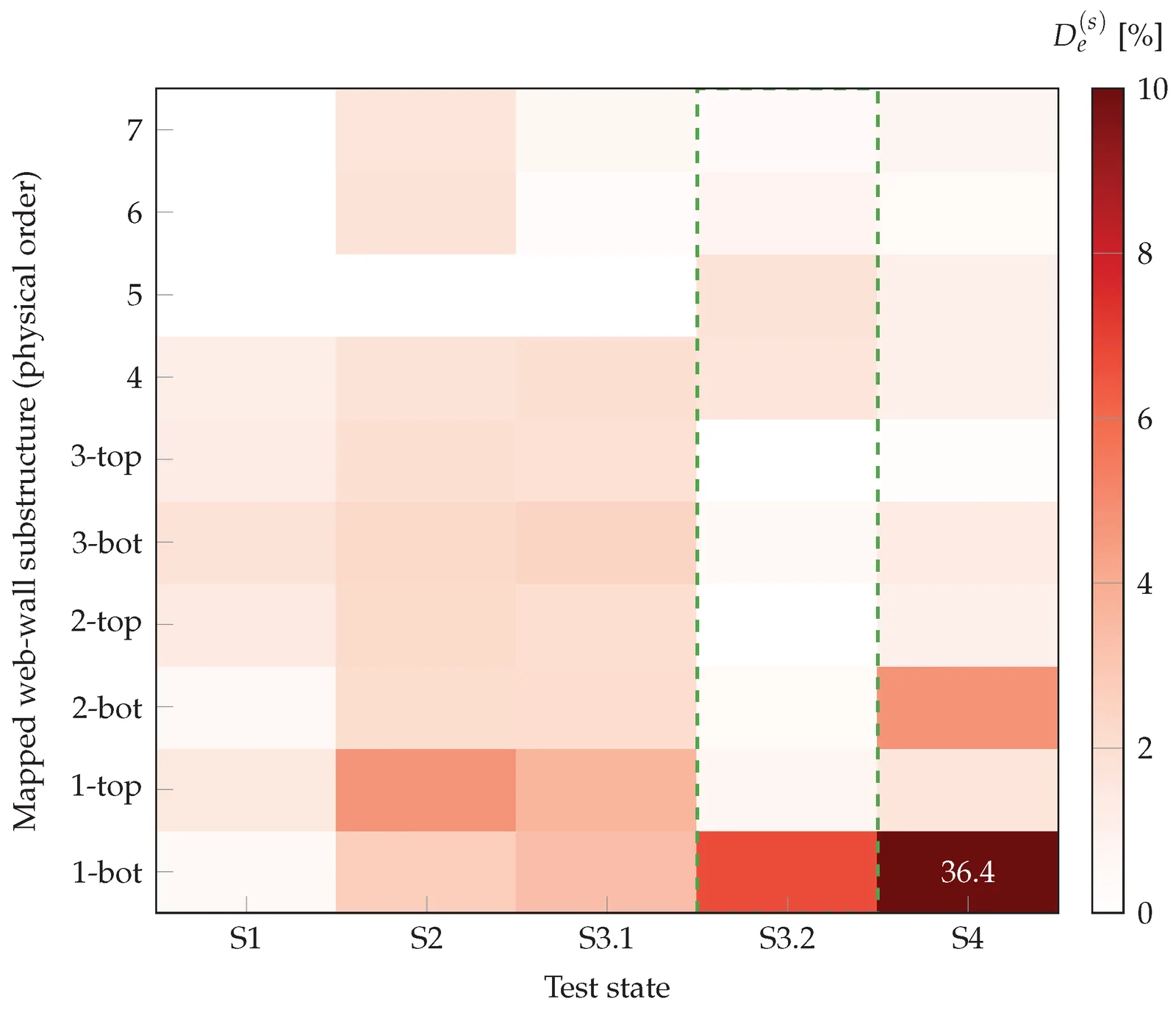
Spatiotemporal heatmap of best method’s edge scores across the ten mapped substructures and five non-baseline states. Rows are ordered physically, with 1-bot at the bottom. The color scale is capped at 10%; the S4/1-bot value (36.4%) is annotated directly. The green border marks S3.2 because it follows brace stiffening rather than a new earthquake input.

Table 3 reports rank agreement as a single number per state, which hides where the agreement is good and where it fails. Figure 5 is a rank diagnostic, not a physical-location plot; it unpacks the Table 3 correlations into the actual rank–rank scatter for each state. Each marker is one of the ten mapped substructures placed at coordinates (paper damage rank, network score rank), with rank 1 denoting the most damaged substructure in the paper and the largest edge-drop score in the network. The four lower-region substructures 1-bot through 2-top use distinct red marker shapes, as shown in the legend, and blue squares denote the remaining upper substructures 3-bot through 7. A marker on the dashed diagonal indicates that the network ranks that substructure at the same position as the paper damage factor; vertical displacement from the diagonal indicates rank disagreement. The Spearman ρ reported in each panel header is, equivalently, the correlation between the x- and y-coordinates of the points. The axes are rank positions rather than physical coordinates, so the markers are not located by floor height; the physical ordering of the same substructure names is shown separately in the heatmap of Figure 6. This figure therefore diagnoses agreement in ordering, while Figure 6 shows where those ordered substructures sit in the wall.

Reading the four panels in sequence makes the limitation at S1 concrete. In the S1 panel, the network ranks 1-bot at position 7 (out of 10), while the FE model ranks it at position 1, a five-rank departure from the diagonal that drives most of the ρ=0.47 shortfall. The remaining S1 markers are also scattered relative to the diagonal, with the paper’s most damaged substructure 1-bot, the network’s top substructure 3-bot, and several mid-rank substructures placed inconsistently between the two methods. At S2 and S3.1, by contrast, the lower-region markers move close to the diagonal, which is why the global rank agreement rises to ρ=0.89 and 0.94. At S4, the network’s top-ranked lower-wall substructures are also consistent with the visual evidence reported by Moaveni et al. [2], which places the dominant physical damage in the bottom two stories. The remaining S4 mismatch is mainly an upper-region rank difference: the paper places 3-top in the middle of the damage list, while the network places it near the bottom. This is less damaging to the overall rank statistic than the S1 1-bot mismatch and is consistent with the earlier observation that the published FE model result itself contains some discrepancy relative to the visual damage pattern.

For the spatiotemporal evolution across substructures and states, Figure 6 replaces the previous per-state bar arrays with a single heatmap. Unlike Figure 5, this figure restores the physical order of the mapped wall substructures. Rows are arranged in physical building order, with the lowest substructure 1-bot at the bottom of the panel and the topmost substructure 7 at the top, so the reader can visually trace where the strongest correlation drops sit in the structure. Columns are the test states; the S3.2 column is bordered in green because it follows the bracing modification discussed in the S3.2 subsection rather than a new earthquake. The color scale is capped at 10% to keep the non-S4 cells legible: the S4/1-bot cell carries the dataset’s largest value (36.4%) and is annotated with its numeric value rather than overwhelming the colormap.

The heatmap consolidates three observations into one image. First, the bottom row 1-bot darkens monotonically from S1 to S4, including across S3.2, with the S4 cell off the color scale; this is the cumulative base-region damage signal that drives the headline S4 result. Second, the S3.2 column is not a faded version of S3.1 nor a stronger version: it has its own pattern, with 1-bot continuing to darken, mid-substructure rows fading toward white, and upper rows 4 and 5 lighting up. This is the brace-change pattern described in the S3.2 subsection; the heatmap representation makes its qualitative difference from the surrounding damage-state columns easy to see at a glance. Third, substructures 5–7 (top three rows) carry near-zero signal at S1, S2, and S3.1, consistent with no damage being expected there in the published paper’s interpretation [2]; their non-zero values at S2 (visible as light cells) sit within the noise floor of the indicator.

### 4.4. Base Sensor Importance

The base edge H0-1–MH-1 carries the largest local signal at S4: a 36.4% correlation-weight drop, roughly eight times larger than the next-largest edge (2-bot, 4.8%). This matches the paper’s physical interpretation that base rotation and bond slip become important in the white-noise identification case. The same location also has the largest S4 centrality loss in Table 2 (Δc=−0.066), so the local edge drop and node-level centrality-change views point to the same base-region disruption. The ablation result is nuanced. Removing the base edge changes the best-method rank correlations from (0.47,0.89,0.94,0.86) to (0.63,0.87,0.93,0.83) at S1, S2, S3.1, and S4, respectively. Two opposite effects therefore coexist on the same edge. At S4, the base edge supplies the clearest local signature of base-region damage; removing it lowers the global rank agreement slightly (0.86 → 0.83) and removes the largest single contribution to the heatmap of Figure 6. At S1, however, the base edge improves the global rank agreement (0.47 → 0.63): the network places 1-bot at rank 7 while the FE model places it at rank 1 (Figure 5), so the base reading is the single largest contributor to the S1 disagreement, and dropping it removes the source of that disagreement.

The base-sensor result is one specific case in which the network surfaces structural information that the FE model analysis does not produce directly. The strongest such case is state S3.2, examined next.

### 4.5. Structural Change at State S3.2

State S3.2 is the state where the proposed indicator remains applicable while the calibrated FE benchmark is not directly applicable. No new earthquake input was applied between S3.1 and S3.2; instead, the diaphragm-to-column bracing was intentionally stiffened to recover the target second-mode frequency, layering a known structural modification on top of pre-existing damage. Because the published FE parameterization does not represent this post-modification configuration, S3.2 has no comparable benchmark damage factor. The correlation graph still provides a direct data-only comparison of which edges strengthened and which continued to weaken, and we interpret this pattern in light of the known brace-stiffening design intent rather than as a pure new-damage step.

**Figure 7:**
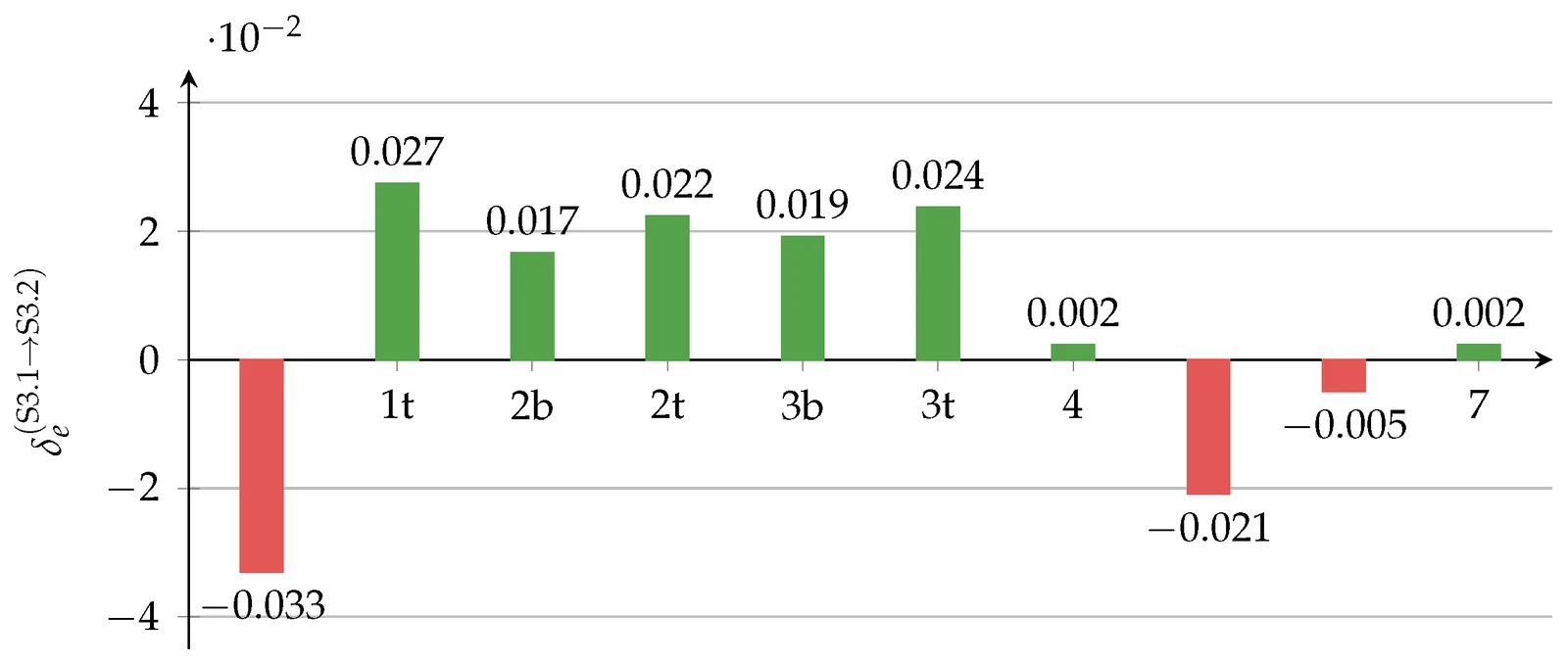
Signed edge weight change from S3.1 to S3.2 on the Spearman absolute correlation graph. Green: edges that strengthen after the brace modification (coupling restored on mid-substructure edges). Red: edges that continue to weaken (base edge and upper edges 5, 6). The pattern is not what a pure damage step would produce; it shows the brace-change effect together with continued base-edge weakening.

Figure 7 summarizes the signed edge weight change $\delta_{e}^{\left(S3.1\to S3.2\right)}$ from Equation (10) for the ten mapped edges on the Spearman absolute correlation graph. Three features stand out.

1. The five mid-substructure edges 1-top, 2-bot, 2-top, 3-bot, and 3-top all show a positive $\delta_{e}$ of comparable magnitude (+0.017 to +0.027), indicating that the bracing restored a portion of the inter-floor coupling lost during prior damage.
2. The base edge 1-bot is the single largest negative entry (−0.033) and is at least 50% larger in magnitude than any other negative entry.
3. Upper edges 4 and 7 are near zero (±0.002), as expected for substructures not directly affected by either earlier damage or the brace modification. Edge 5 shows a moderate negative change (−0.021) and edge 6 a weak one (−0.005), consistent with the load-path change discussed below.

A pure damage progression would generally produce negative $\delta_{e}$ values, particularly near newly damaged regions. S3.2 shows a different pattern: several mid-substructure edge correlations increase, while the base-edge correlation continues to decrease. Because S3.2 represents a diaphragm-bracing modification rather than a new damage state, this pattern indicates a redistribution of the structural response. However, correlation measurements alone cannot determine whether the base-edge reduction reflects additional damage or response redistribution; therefore, it is not interpreted as direct evidence of new base damage. This interpretation is consistent with the S0-referenced heatmap, which shows the cumulative change from the undamaged state.

**Figure 8:**
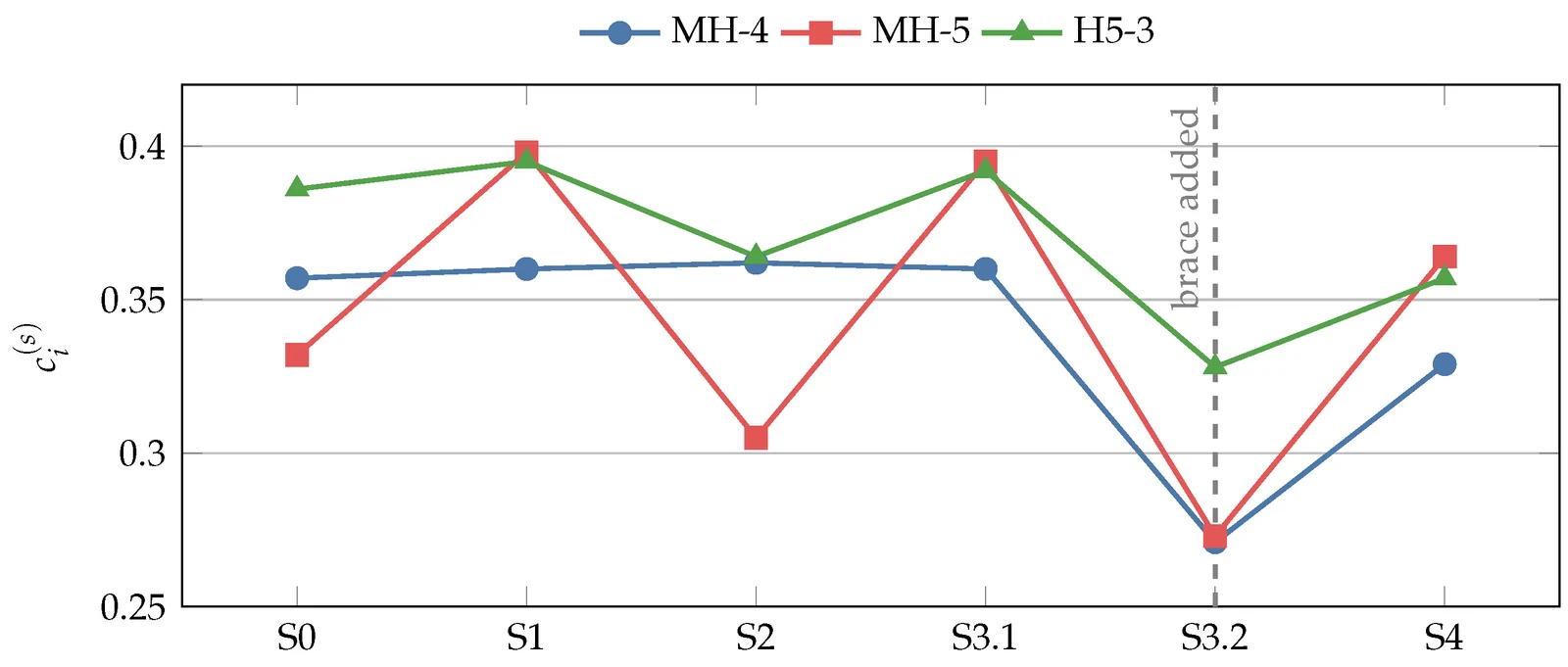
Mid-floor sensor centrality trajectory across states. MH-4 and MH-5 lose roughly 24% and 18% of their thresholded weighted-degree centrality at S3.2 and partially recover at S4; an off-web sensor on the same floor (H5-3) shows the same qualitative excursion. The S3.2 dip is consistent with the added bracing locally constraining the mid-floor sensors, so that they decouple from the global response while the brace dominates them.

The same physical picture appears in the centrality view (Figure 8). Mid-floor sensors MH-4 and MH-5, which sit close to where the bracing acts, lose about 24% and 18% of their thresholded weighted-degree centrality at S3.2 (0.357→0.271) and (0.332→0.273), respectively, and partially recover by S4. An off-web sensor on the same floor (H5-3) shows a smaller but similar excursion. The interpretation is that the added bracing locally constrains mid-floor motion: those sensors are then driven more by the brace and less by the rest of the structure, which lowers their graph coupling. When a subsequent earthquake input at S4 either reduces the effectiveness of the brace or damages adjacent regions, the mid-floor sensors partially rejoin the global response, and their centrality moves back toward the baseline. This is a graph-level diagnostic of the brace–structure interaction that is not directly visible in the FE model damage-factor table.

Centrality-change localization is therefore best treated as a secondary check rather than as a replacement for mapped edge drops. Aggregating positive centrality losses by floor, the bottom floors 0–2 account for 0.61, 0.41, 0.80, 0.12, and 0.39 of the total loss at S1, S2, S3.1, S3.2, and S4. The large S3.1 lower-story share is consistent with the damage pattern, while the small S3.2 share reflects that the brace modification perturbs the mid floors instead of acting like a new bottom-story damage step.

In short, S3.2 is the state at which the network indicator is informative beyond what the FE model benchmark can supply. The benchmark FE model reference cannot rank S3.2 substructure damage because its parameterization does not represent the modified structure. The network reports, in one map, that the bracing restored coupling on the mid-substructure edges, that the base edge continued to weaken (read as response redistribution, not new base damage), and that mid-floor sensors were locally constrained by the brace. This combination of observations can be obtained directly from the measured response records, without requiring an updated FE parameterization, an identified mode set, or supervised training data.

### 4.6. Network-Informed RDT as a Secondary Check

The same spatial-correlation graph that localizes coupling drops also supplies a sensor-selection rule for output-only modal-frequency extraction. Sensors with high S0 centrality participate strongly in the dominant longitudinal response, which makes them natural reference signals for signal processing like RDT extraction. RDT is a non-parametric time-domain method that forms an approximate free-decay signature by averaging short response segments triggered when the signal crosses a prescribed amplitude threshold.

Choosing the trigger reference sensor for the multi-channel RDT is always an open question [38,39]. Then, the concept of centrality helps to define a principled sensor-selection rule that identifies sensors with strong coupling to the global response.

**Figure 9:**
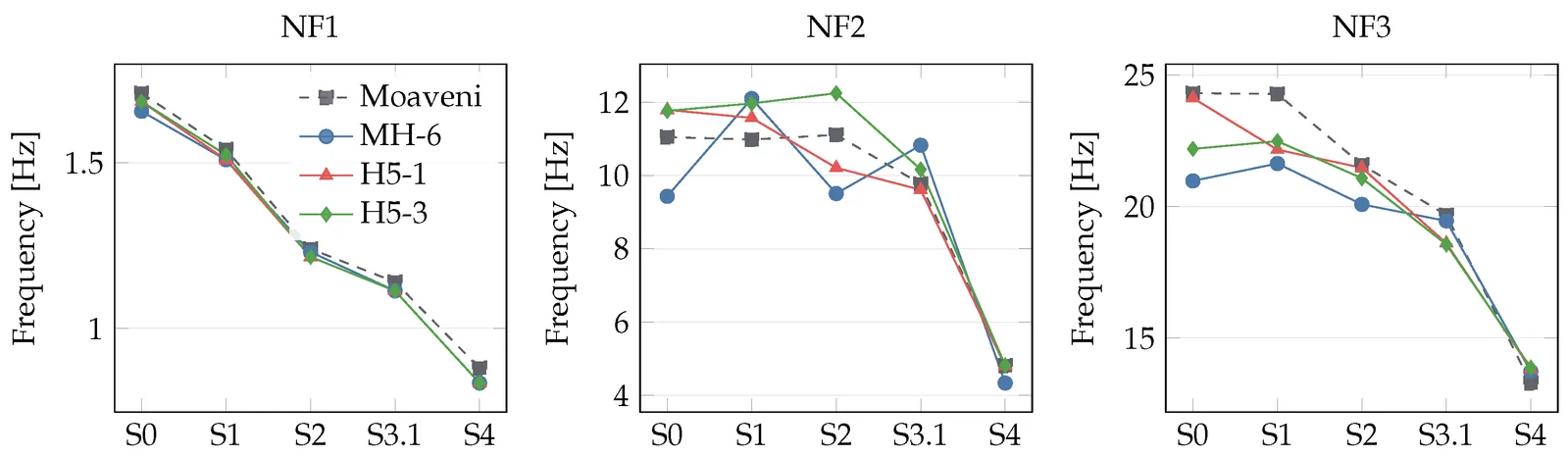
First three natural frequencies vs. damage state, comparing the published 0.03 g white-noise modal estimates of Moaveni et al. [2] (dashed gray) with 4 s RDT estimates triggered from each of the top-three S0-central sensors selected by the correlation graph (MH-6, H5-1, H5-3). The fundamental frequency NF1 (left) softens monotonically from approximately 1.7 Hz at S0 to approximately 0.85 Hz at S4, a drop of roughly 50% that all three reference sensors track to within a few percent of the paper. NF2 (middle) and NF3 (right) are more sensitive to the trigger reference; H5-1 is consistently the best of the three central sensors.

Using the top S0 central sensors as trigger references, the resulting multi-channel RDT spectra recover the first three natural frequencies in good agreement with the published 0.03 g white-noise values: as Figure 9 shows, the network-triggered RDT estimates closely track the paper’s frequencies across all damage states, with mean absolute errors of approximately 3–5.5% for the best central reference, and the fundamental frequency follows the full ≈50% softening from 1.68 Hz to 0.84 Hz (Table 5).

This does not make the correlation network a modal identifier. Rather, the network selects high-coupling sensors for response averaging, while RDT extracts global modal frequencies. This distinction is useful: edge drops localize changes, while RDT frequencies document global softening.

The value of this check is the sensor selection itself, not the recovered frequencies, which the published FE model reproduces by construction. The three triggers used here (MH-6, H5-1, H5-3) are exactly the top-three sensors of the 29-channel S0 weighted-degree centrality ranking (Table 2), and they appear as the largest nodes in the 3D correlation network of Figure 3. Selecting them requires neither an identified mode set nor a calibrated model, in contrast to the benchmark analysis, which identifies the modes from a fixed set of 14 longitudinal wall channels and then updates a 17–18-substructure finite element model [2], as well as to hybrid output-only identification on the same specimen, which couples random decrements with Kalman estimation of the full system matrices [40]. The selection is physically plausible, but it should not be interpreted as a direct proxy for first-mode displacement amplitude.

Here, centrality is defined as the thresholded weighted degree computed from broadband pairwise response correlations. It therefore favors sensors with many strong network connections, rather than sensors with the largest first-mode amplitudes. The floor 5–6 web and mid-height channels can consequently outrank the roof channels because they remain strongly coupled to both the lower and upper stories, whereas the roof sensors have fewer above-threshold connections. We therefore interpret the floor 5–6 maximum as a network-coupling result consistent with dominant longitudinal participation, without equating it directly with the peak first-mode amplitude. Thus, the same spatial-correlation graph used for damage localization can also guide reference-sensor selection based on the measured structural response.

## 5. Discussion

The central message of this study is that the spatial coordination of a structure’s measured responses is itself a usable structural-dynamics feature: damage and other physical changes leave a signature in how the sensor signals move together, and that signature can be read directly from broadband response records without first updating a finite element model, identifying modes, or training a classifier. This reframes vibration-based damage localization as a question about the measured responses themselves rather than about a model fitted to them, and the practical advantages below follow from that shift.

This approach offers four advantages over a model-based pipeline. First, it is prior-light: it does not require a finite element model, an identified mode set, or labeled training data to construct the network indicator, so it can be applied directly to a new structure from its raw ambient or white-noise records. Second, it stays usable where a model-based pipeline must skip; the brace-stiffening state S3.2 has no calibrated parameterization and therefore no benchmark damage factor, yet the method still returns an internally consistent reading of which regions recouple and which continue to weaken. Third, the same response-coordination view supplies a principled, data-driven rule for choosing reference sensors: the most strongly coupled channels recover the published natural frequencies in close agreement (Figure 9), turning sensor selection from a modeling assumption to a measured property of the response. Fourth, the diagnostic is transparent and inexpensive; every step is a correlation, a comparison against a baseline, and a mapping back to physical regions. So, an engineer can audit why a region is flagged, in contrast to high-capacity learned models, whose decisions are harder to inspect.

For the present dataset, the core workflow processed all six eight-minute, 29-channel records in a median total time of 1.014 s over seven repeated runs, or 1.927 s when data loading was included. This timing covers correlation network construction, damage-score calculation, and sensor-centrality evaluation, but excludes plotting, RDT analysis, and offline robustness tests. Unlike iterative FE model updating, the core workflow requires no mesh construction, repeated eigensolutions, sensitivity matrices, or parameter optimization. For $n_{s}$ sensors and *N* samples, correlation network construction scales approximately as $O(n_{s}^{2}N)$ in computation and $O(n_{s}^{2})$ in storage; the present timing supports rapid batch screening, although real-time operation has not yet been demonstrated. Because the benchmark FE study does not report its runtime, hardware, or iteration count, this comparison is qualitative rather than a direct wall-clock speedup measurement.

The method has several limitations that bound its present scope. It is weak to light damage, where the true changes are small and several regions are nearly tied. It is a localization screen rather than a quantification tool: it ranks where coordination changes but does not estimate physical damage severity or stiffness loss. The method also requires a structure-specific reference condition; for an existing structure, this reference can be established when monitoring begins, but subsequent scores identify change relative to that condition rather than damage relative to a certified pristine state. The present scalar network uses only longitudinal channels and zero-lag correlations, so it does not explicitly represent multidirectional responses, phase relationships, time delays, or nonlinear dependence. The demonstrated scope is therefore limited to one structure and its excitation program. Finally, the stability of centrality rankings under different loading conditions, sensor layouts, and environmental variability has not yet been established.

Several extensions follow naturally from the present limitations. First, uncertainty-aware operational baselines and confidence bounds on edge weight changes should be developed for online monitoring under environmental and operational variability. Second, frequency-dependent, time-lagged, and nonlinear dependence measures, including band-limited coherence around individual modes, should be evaluated to improve sensitivity to light damage and distinguish phase, delay, and nonlinear interactions. Third, vector-valued graph signals should be investigated to combine longitudinal, transverse, and vertical responses without losing their directional structure. A physics-informed definition of response coordination could further weight pairwise measures with mechanical priors, providing a path from localization toward damage quantification and a tighter link to finite element updating. The stability of centrality rankings under changing loading, sensor-layout, and environmental conditions should be systematically evaluated, and centrality-based criteria should be extended to optimized sensor placement, automated spatial mapping, and data fusion in output-only identification. Finally, validation on additional structural systems, sensor layouts, excitation types, and environmental conditions is needed to establish the framework’s generality.

## 6. Conclusions

This study proposed a spatial-correlation sensor network that provides a transparent, data-driven front-end for damage localization, sensor selection, and structural-reconfiguration screening, complementary to FE updating and modal identification. The principal conclusions of this study are summarized as follows:

1. This study represented the instrumented UCSD seven-story shear wall as a sensor network, in which the accelerometers are graph nodes and the pairwise spatial correlations of their responses are edge weights. Changes in these edge weights relative to the undamaged baseline were used to localize damage without first updating a finite element model, identifying modal parameters, or training a supervised classifier. Across the different damage scenarios, the spatial-correlation network was able to localize the induced damage. The selected indicator agrees well with the benchmark FE model in their localization pattern at the moderate- and severe-damage states, while the mild-damage state stays close to the baseline variability, so the method is best read as a practical screening tool rather than a stiffness-loss estimator.
2. The network proved especially valuable for the structure reinforcement and repair state, where the FE reference cannot be applied directly because its calibrated parameterization no longer represents the modified structure. The network localized the mid-height recoupling and a response redistribution rather than a pure damage progression, providing complementary information that is not represented in the FE model, and doing so without performing iterative FE model updating. Network centrality reinforced this reading: it exposed the brace–structure interaction as a localized constraint on the mid-floor sensors, which decoupled from the global response and then partially rejoined it once subsequent shaking acted, a graph-level diagnostic absent from the FE model. The same baseline centrality also singled out the most informative sensors, which recovered the benchmark modal-frequency trend through random decrement analysis.
3. This correlation-network-based structural monitoring approach still presents open questions requiring further investigation, particularly for early damage detection and the quantification of uncertainty and damage severity. Future research will address these limitations through uncertainty-aware baselines, richer dependence measures, multidirectional and physics-informed graph formulations, optimized sensor placement and spatial mapping, and validation across additional structures and operating conditions.

## Figures and Tables

**Table 1:** UCSD damage-state sequence used in this study. Test numbers follow the public UCSD data description; only the 0.03 g white-noise portions are analyzed here. The right column summarizes the published FE model reference pattern used for comparison [2].

State	Test	Preceding Action	Published FE Model Top-3 Substructures (Ranked)
S0	39	Baseline white-noise/ambient-vibration test	— (reference state)
S1	41	After EQ1	1-bot, 2-bot, 1-top (mild lower-wall damage)
S2	46	After EQ2	1-bot, 1-top, 2-bot (lower two stories)
S3.1	49	After EQ3	1-bot, 1-top, 2-bot (accumulated lower-story damage)
S3.2	61	Brace stiffened after S3.1	— (no comparable FE damage factors reported)
S4	64	After EQ4	1-bot, 1-top, 2-bot (severe; top three tied at ≈92%)

**Table 2:** Most central S0 sensors and largest centrality losses at S4. Centrality identifies useful references; centrality change is the quantity relevant to damage localization.

Sensor Centrality at S0			Sensors with the Largest Centrality Losses at S4		
Sensor	Floor	$c_{i}^{\left(0\right)}$	Sensor	Floor	$\Delta c_{i}^{\left(S4\right)}$
MH-6	6	0.399	H0-1	0	−0.066
H5-1	5	0.395	H3-2	3	−0.060
H5-3	5	0.386	H2-1	2	−0.056
H6-1	6	0.369	H7-2	7	−0.034
MH-4	4	0.357	MH-6	6	−0.032
MH-5	5	0.332	H5-3	5	−0.029
H4-1	4	0.330	MH-4	4	−0.028
H6-3	6	0.325	H7-3	7	−0.003

**Table 3:** Rank agreement for Spearman absolute correlation with percent-drop scoring. *p*-values are two-sided Monte Carlo permutation values with n=10 substructures and 200,000 random permutations. The S1 row is consistent with the null at the 5% level; the remaining three states are significant.

State	ρ vs. Paper	Perm. *p*	Network Top Three	Paper Top Three
S1	0.47	0.175	3-bot, 1-top, 2-top	1-bot, 2-bot, 1-top
S2	0.89	0.00094	1-top, 1-bot, 3-bot	1-bot, 1-top, 2-bot
S3.1	0.94	0.00028	1-top, 1-bot, 3-bot	1-bot, 1-top, 2-bot
S4	0.86	0.00262	1-bot, 2-bot, 1-top	1-bot, 1-top, 2-bot
Mean (S1–S4)	0.79	–	–	–
LOOCV mean	0.76	–	–	–
LOOCV: Leave-one-state-out cross-validation; the indicator is selected using three damage states and evaluated on the held-out state.				

**Table 4:** Robustness diagnostics for the best indicator. The digitization intervals are 2.5–97.5% quantiles after perturbing the visually digitized FE damage factors by ±5 percentage points. The baseline column counts mapped edges out of ten, whose state weight falls below the lower 95% S0 block interval estimated from sixteen 30 s S0 windows.

State	ρ Digitization Interval	Edges Below S0 CI/10
S1	[0.08, 0.52]	3/10
S2	[0.75, 0.94]	6/10
S3.1	[0.84, 0.95]	6/10
S4	[0.76, 0.88]	4/10

**Table 5:** Natural frequencies from the paper’s 0.03 g white-noise tests and best network-triggered RDT estimates using the current 4 s RDT window.

State	Ref.	Trig.	NF1	NF2	NF3
S0	H5-1	89	1.68/1.71	11.79/11.05	24.13/24.31
S1	H5-1	91	1.51/1.54	11.57/10.98	22.16/24.28
S2	H5-1	94	1.22/1.24	10.21/11.11	21.47/21.59
S3.1	H5-1	85	1.11/1.14	9.61/9.77	18.60/19.68
S4	H5-1	80	0.84/0.88	4.80/4.81	13.83/13.29

Entries are RDT/paper frequencies in Hz; “Trig.” is the number of accepted 1.4σ RDT trigger windows. The mean absolute frequency error for these best central references is 3.0, 5.4, 3.5, 3.2, and 3.1% from S0 to S4.

## Data Availability

The analysis code is publicly available at https://github.com/cisgroup/training-free-spatial-sensor-network (accessed on 1 August 2026).
